# Dissociated Grey Matter Changes with Prolonged Addiction and Extended Abstinence in Cocaine Users

**DOI:** 10.1371/journal.pone.0059645

**Published:** 2013-03-18

**Authors:** Colm G. Connolly, Ryan P. Bell, John J. Foxe, Hugh Garavan

**Affiliations:** 1 Department of Psychiatry, University of California San Francisco, San Francisco, California, United States of America; 2 Department of Psychology, The City College of the City University of New York, New York, New York, United States of America; 3 Departments of Pediatrics and Neuroscience, Albert Einstein College of Medicine, New York, United States of America; 4 The Cognitive Neuroscience and Schizophrenia Research Program, The Nathan S. Kline Institute for Psychiatric Research, New York, United States of America; 5 School of Psychology and Institute of Neuroscience, Trinity College Dublin, Dublin, Ireland; 6 Departments of Psychiatry and Psychology, University of Vermont, Burlington, Vermont, United States of America; Yale University School of Medicine, United States of America

## Abstract

Extensive evidence indicates that current and recently abstinent cocaine abusers compared to drug-naïve controls have decreased grey matter in regions such as the anterior cingulate, lateral prefrontal and insular cortex. Relatively little is known, however, about the persistence of these deficits in long-term abstinence despite the implications this has for recovery and relapse. Optimized voxel based morphometry was used to assess how local grey matter volume varies with years of drug use and length of abstinence in a cross-sectional study of cocaine users with various durations of abstinence (1–102 weeks) and years of use (0.3–24 years). Lower grey matter volume associated with years of use was observed for several regions including anterior cingulate, inferior frontal gyrus and insular cortex. Conversely, higher grey matter volumes associated with abstinence duration were seen in non-overlapping regions that included the anterior and posterior cingulate, insular, right ventral and left dorsal prefrontal cortex. Grey matter volumes in cocaine dependent individuals crossed those of drug-naïve controls after 35 weeks of abstinence, with greater than normal volumes in users with longer abstinence. The brains of abstinent users are characterized by regional grey matter volumes, which on average, exceed drug-naïve volumes in those users who have maintained abstinence for more than 35 weeks. The asymmetry between the regions showing alterations with extended years of use and prolonged abstinence suggest that recovery involves distinct neurobiological processes rather than being a reversal of disease-related changes. Specifically, the results suggest that regions critical to behavioral control may be important to prolonged, successful, abstinence.

## Introduction

Cocaine is a major worldwide public health issue for which current treatments are unsatisfactory [1], [2]. Understanding the differences between the brains of cocaine users and nonusers is a critical step in identifying neurobiological characteristics of addiction that may guide the development of therapeutic interventions. Also of considerable importance, but much less well researched, is understanding what differentiates users who abstain and successfully avoid relapse from those who fail to maintain abstinence and repeatedly relapse. As treatment programs typically have very high dropout rates [3], [4] reflecting the relapsing nature of the disease, an understanding of the neurobiology of successful abstinence may identify key targets for therapeutic interventions. However, one consequence of high dropout rates is that little is known about the neurobiology of successful long-term abstinence as high levels of relapse and attrition from treatment makes prospective studies of long-term abstinence effects difficult.

Voxel based morphometry [5] is a technique that can examine local tissue volume differences. Using this method, relative to healthy drug-naïve controls, grey matter changes have been observed in multiple regions of the brain of cocaine addicts. Widespread decreased GM concentration has been reported in lateral and medial aspects of the orbitofrontal cortex (OFC), anterior cingulate (ACC), anteroventral insular cortices, lateral prefrontal cortex (LPFC), temporal cortices [6]–[11], cerebellum [12] and subcortical regions [13]–[15]. Cocaine use has been linked to accelerated age-related decreases in grey matter in the temporal lobes [16]. Fein et al. [17] using a related method observed significant reduction in prefrontal grey matter volume for cocaine dependent (CD) and combined cocaine and alcohol dependent individuals. It has been suggested that these focal decreases in GM may underlie the functional hypoactivity and cognitive deficits observed in cocaine users [8]. These regions have been variously implicated in the executive functions of conflict monitoring [18], performance monitoring [19], interoception [20], decision-making [21] and reward processing [22], all of which have been demonstrated to be compromised in cocaine addicts. However, the literature is not consistent as others have failed to observe differences in GM between CD and control participants [23].

Our prior report characterizing long-term abstinence probed the functional neuroanatomy of cognitive control using a GO/NOGO task [24]. The short- and long-term abstinent CD groups in this study displayed greater activation levels for correct inhibitions and errors relative to drug-naïve controls. More specifically, the results suggested that early abstinence (1–5 weeks) may be characterized by heightened activity in regions subserving inhibitory control with heightened activity underlying behavioral monitoring processes playing a more prominent role later in abstinence (40–102 weeks). Our previous investigation of white matter using diffusion tensor imaging revealed one set of structural changes that differentiated long-term abstinent (44–102 weeks) from more recently abstinent users (1–5 weeks) and another set that differentiated all abstinent individuals from healthy controls [25]. One interpretation is that the first set of white matter changes may arise during abstinence or may have preceded and facilitated abstinence while the second set may reflect changes that arose from or preceded cocaine use. An implication arising from this interpretation is that abstinence and recovery may have neurobiological underpinnings that are distinct from those associated with the disease.

A recent study compared grey and white matter densities in abstinent (1–16 weeks) and current CD individuals and healthy control participants and observed that the current users, compared to controls and abstainers, had lower tissue density in frontal, temporal, cerebellar and subcortical regions. The abstinent group had much less pronounced deficits with lower grey matter density in caudate/putamen and bilateral cerebellum compared to controls [13]. It would appear that GM deficits are reduced in abstinent users but it remains unclear whether these differences would persist with prolonged abstinence, due in part to the high rates of relapse making such prospective studies difficult.

The aim of the present study, using a cross-sectional design, was to examine volume differences in cortical grey matter in a sample of former cocaine addicts who varied in length of abstinence and duration of use. We hypothesized that abstinence duration would be associated with a set of GM volume changes in regions critical to executive function, specifically anterior cingulate and lateral prefrontal cortex. We further hypothesized that any GM volume changes that may be attributable to length of use would be distinct from those related to abstinence duration. Comparison to a non-drug using control group allowed us to assess how changes of GM with abstinence duration relate to volumes typical of drug naïve controls. The cross-sectional design employed here suffers by being unable to resolve whether effects related to abstinence duration arose from abstinence or preceded abstinence. However, it is nonetheless valuable in that it can characterize individuals with a demonstrated ability to remain abstinent over various durations. This characterization may be of therapeutic importance in that observed neurobiological differences might serve as targets for therapy. Additionally, they may be useful biomarkers for possible investigation in future longitudinal studies of abstinence.

## Materials and Methods

### Ethics Statement

This study was approved by the Institutional Review Board of the Nathan S. Klein Institute for Psychiatric Research (NKI).

### Participants

Eighty-six volunteers (9 female; mean age 38.1, range 20–55) (see Table 1) participated in this study. Written informed consent was obtained in accordance with the Declaration of Helsinki and participants were compensated for their time. Participants were divided into two groups: one group of 43 abstinent cocaine users (2 female) and a second of 43 age-matched controls (7 female). Control participants were recruited from the volunteer recruitment pool at the NKI. CD participants were recruited from in-patient and out-patient treatment centers in New York State. All CD participants received an initial diagnosis of cocaine dependence as assessed by Structural Clinical Interview for the DSM-IV (SCID) [26]. Participants early in treatment were in an in-patient facility that was monitored on a 24-hour basis. They were subject to periodic Breathalyzer tests for alcohol and random urine toxicology screens for multiple substances. Additionally, subjects were not permitted to leave the facility without an escort. Those later in treatment were allowed to leave the facility on their own recognizance but were evaluated by clinical staff (including urine toxicology and Breathalyzer tests) upon their return. Continued enrollment in the in-patient and out-patient treatment programs was predicated on negative toxicology screenings. CD participants met at least weekly with a personal counselor certified by the state of New York in the treatment of alcoholism and drug abuse. Length of abstinence was verified with the counselor at the addiction treatment centers. Exclusion criteria for both CD and control participants were: (1) Any DSM IV, Axis 1 diagnosis excluding dependence or a past diagnosis of depression caused by CD based on the SCID; (2) Head trauma resulting in loss of consciousness for longer than 30 minutes; (3) Presence of any past or current brain pathology; (4) A HIV diagnosis; (5) Contraindications for MRI; (6) Under 19 or over 55 year of age; (7) The presence of white matter (WM) hyperintensity (only one patient was excluded due to clinically significant WM hyperintensity). Given the high rates of co-morbid alcohol and drug abuse in the target patient population [27], participants were not excluded for abuse of other drugs or alcohol prior to the onset of CD (3 participants had co-morbid alcohol dependence and 7 had co-morbid heroin dependence.) Thus the CD group may be thought of as polydrug abusers with a primary dependence on cocaine. None were currently using any amount of alcohol or drugs. Years of drug use prior to abstinence was obtained during the initial SCID interview.

**Table 1:** Demographic characteristics for the control and abstinent cocaine groups.

Characteristic	Controls	Cocaine Dependent
Number of participants	43	43
Gender M/F	41/2	36/7
Age at time of scanning(years)	38.7±1.6 (20–55)	37.5±1.2 (22–54)
Years of education	14.6±0.3 (12–20)	12.3±0.3 (7–17) ***
Abstinence (Weeks)	Not Applicable	31.25±4.5 (0.7–102)
Years of use	Not Applicable	9.1±1.0 (0.3–24)

Entries are of the form: mean ± SEM (min-max). Gender was compared using the binomial proportion test; all other observations were compared by Welsh T-tests. C = control, CD = cocaine dependent. Significance code: p ≤ 0.001 ‘***’.

### MR Data Acquisition

All scanning was conducted on a 1.5T Siemens VISION scanner (Erlangen, Germany) at NKI that was equipped with a 30.5-cm i.d. three-axis local gradient coil and an end-capped quadrature birdcage radio-frequency head coil. High-resolution T1-weighted MPRAGE anatomical images were acquired with the following parameters: TE = 4.9 ms, TR = 11.6 ms, flip angle 8°, FOV 300 mm, 1.2 mm isotropic voxels, matrix 256×256, and 172 sagittal slices.

### MR Data Analysis

The high-resolution T1-weighted images were subjected to a voxel-based morphometry (VBM) analysis [5], [28] carried out with FSL tools [29]. The data were median filtered (3×3 voxels), brain-extracted using AFNI’s 3dSkullStrip [30], and then segmented into grey and white matter and cerebrospinal fluid [31]. The grey matter images were then affinely aligned to MNI152 standard space [32], [33] followed by non-linear registration [34], [35] to further refine the alignment. The resulting data were averaged to create a study-specific template, to which the native grey matter images were then non-linearly re-registered. The registered partial volume images were then modulated by multiplying by the Jacobian of the warp field [28]. This step compensates for the contraction/enlargement due to the non-linear component of the transformation (http://dbm.neuro.uni-jena.de/vbm/segmentation/modulation/), making correcting for total intra-cranial volume of the individual unnecessary [36]. Removal of global brain volume effects in this manner permitted inference on the local GM volume differences. The modulated segmented images were then smoothed with an isotropic Gaussian kernel (σ = 2 mm ∼ 4.7 mm FWHM).

The resultant grey matter images of the abstinent CD group were then subjected to voxelwise Huber robust regression [37], [38] in the R statistical analysis package [39]. The two variables of interest, weeks of abstinence and years of use prior to abstinence were included in a single voxelwise whole-brain regression model. Since years of use could be a proxy for age and given the well-established relationship between age and GM volume [28], [40], age was also included as a nuisance covariate in the regression model. The voxelwise regression coefficients and associated T statistics for each regression term were then split into maps of positive and negative coefficients. Significant voxels passed a voxelwise statistical threshold (t(39) = 2.97, p = 0.005, uncorrected) and, to control for multiple comparisons, were required to be part of a cluster of no less than 360 µl. The volume threshold was determined by a Monte-Carlo simulation that together with the voxelwise threshold resulted in a 5% probability of a cluster surviving due to chance. Regions of interest (ROI) were identified in this manner and the grey matter volume for each region was extracted for each of the CD and, for comparison, the control participants. To determine at what point the GM volume in each region of interest crosses that of the controls, a robust regression line against duration of abstinence and years of use for the CD individuals was fit to these values for each region of interest and the intersection of this line with that of the mean of the controls computed. However, this approach tends to inflate correlation values [41] so care in interpreting the results is warranted.

## Results

### Demographics

The CD participants did not differ from controls in age (Welch t(77.5) = −0.6, p>0.05, or gender (χ^2^ = 1.98, p = 0.15), but did differ on years of education (Welch t(82.6) = −5.1, p<0.001; see Table 1 for demographic information). Years of education correlated negatively with abstinence duration (Pearson’s ρ = −0.43, t(41) = −3.1, p<0.005) but not with years of use (Pearson’s ρ = −0.02, t(41) = −0.12, p>0.1) for the CD group. Years of use did not correlate with length of abstinence (Pearson’s ρ = −0.17, t(41) = −1.2, p>0.05).

### VBM Regression Results

#### Years of use

**Figure 1:**
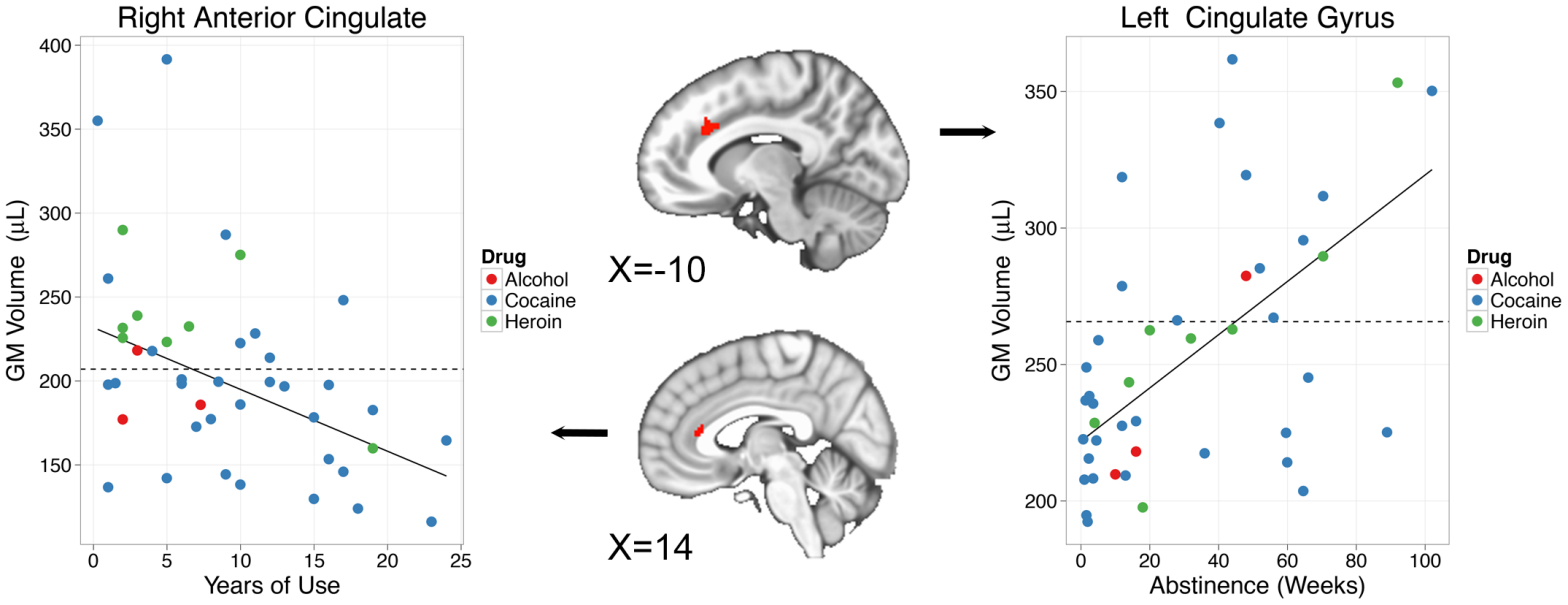
Regions in the left and right anterior cingulate showing, respectively, increases in GM with weeks of abstinence and decreases in GM with years of use. The solid line is the robust regression line for CD individuals. The dashed line is the mean GM in the same ROI for the control participants.

Four regions (Table 2) showed positive correlations with years of use, that is grey matter volume increased in these regions with longer terms of use. These regions were located bilaterally in the precentral gyrus, and one region in each of the left medial frontal gyrus and right nodule of the cerebellum. Several regions (Table 2) displayed negative correlations with years of use. These were located in the right cerebellar tonsil, bilaterally in the superior temporal and inferior frontal gyri, in the right anterior insula, and one in each of the right subcollasal gyrus and right anterior cingulate gyrus shown in Figure 1 (left).

**Table 2:** Regions identified in the regression analysis.

Structure	Hemisphere	BA	Volume	Center of Mass			Crossover
			(µL)	X	Y	Z	Point
**Term: Years of Use Correlation: Positive**							
Precentral Gyrus	L	4	896	−36	−16	62	14.1
Nodule (Cerebellum)	R		888	4	−42	−38	6.2
Medial Frontal Gyrus	L	11	392	−2	32	−14	19.5
Precentral Gyrus	R	6	368	13	−26	73	18.6
**Term: Years of Use Polarity: Negative**							
Cerebellar Tonsil	R		1976	39	−56	−53	4.6
Portions of STG, IFG, AI	R	22/13	1360	49	11	−5	2.8
Portions of STG, IFG	L	22/13	1088	−50	13	−7	3.7
Subcallosal Gyrus	R	34	624	12	1	−17	8.3
Anterior Cingulate	R	24	448	4	30	14	6.7
**Term: Abstinence (weeks) Polarity: Positive**							
Insula	L	13	1512	−37	9	11	29.9
Cuneus	L	31	1480	−26	−79	23	26.4
Superior Frontal Gyrus	L	8	680	−16	46	42	42.3
Culmen	L		648	−14	−48	−12	32.8
Precuneus	R	19	592	31	−78	22	38.0
Cingulate Gyrus	L	32	520	−10	25	31	44.9
Superior Frontal Gyrus	R	10	512	24	54	5	37.1
Cingulate Gyrus	R	31	384	14	−32	41	33.3
**Term: Abstinence (weeks) Polarity: Negative**							
Cuneus	R	19	520	12	−81	36	27.6
Precuneus	L	7	488	−4	−62	57	26.6
Culmen of Vermis	L		368	−2	−64	3	18.5

Center-of-mass coordinates are in the MNI152 (LPI) standard and structure labels are from the Talairach & Tournoux atlas. RL: Right-Left, AP: Anterior-Posterior, IS: Inferior-Superior. STG: Superior Temporal Gyrus, IFG: Inferior Frontal Gyrus, AI: Anterior Insula. BA: Brodmann Area. Crossover point refers to the location on the x-axis (years of use or weeks of abstinence) where the regression (solid) line for the CD users intersects with the mean of the control participants (dashed line). Term refers to the term of interest in the regression model (years of use or weeks of abstinence) from which the clusters were derived. Polarity refers to the sign (positive or negative) of the regression coefficients from which the cluster was generated.

#### Weeks of abstinence

**Figure 2:**
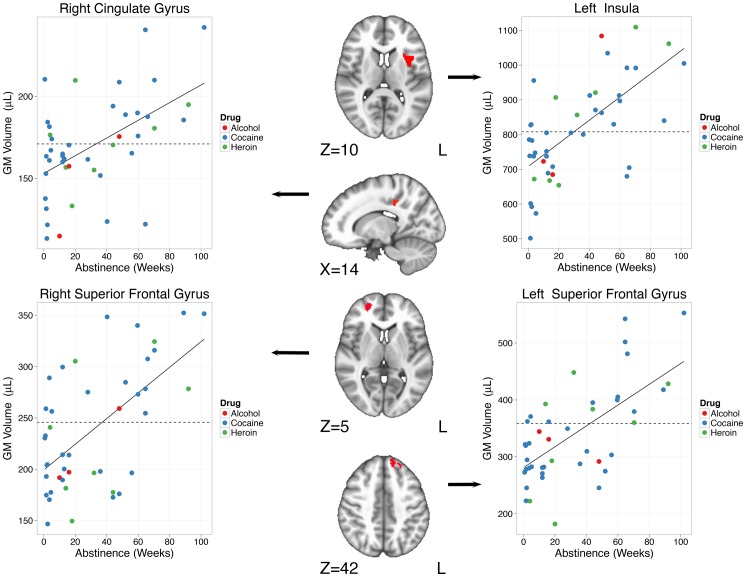
Regions in the right posterior cingulate, left insula and left and right superior frontal gyrii showing increased GM with weeks of abstinence. The solid line is the robust regression line for CD individuals. The dashed line is the mean GM in the same ROI for the control participants.

A number of regions (Table 2) were observed to show positive correlations with weeks of abstinence, that is grey matter volume in these regions increased with abstinence. These included left insula, left and right cingulate gyri, the left cuneus, left and right superior frontal gyri, left culmen of the cerebellum, and the right middle temporal gyrus. As can be seen in Figures 1 and 2, in each of these regions, those CD users with shorter periods of abstinence show less GM than controls. Those who were abstinent longer show greater GM volumes than controls. The cross-over point from relatively smaller to relatively greater volumes was quite consistent across all regions, averaging 35.6 weeks of abstinence (range 26.4–44.9, sd 6.2). Three regions (see Table 2) were observed to display negative correlations with length of abstinence. These included regions in bilateral cuneus and one in the left precuneus. In these regions, on average 24.2 weeks of abstinence (range 18.5–27.6, sd 5.0) passed before the level of GM equaled that of controls and then declined further with increased periods of abstinence.

As abstinence duration correlated with years of education, we conducted cluster-level correlations between GM volumes and weeks of abstinence with both age and years of education included as nuisance regressors. The effects reported above remained significant for all regions.

We conducted a series of Welch T-tests to determine if the GM volumes of users who were abstinent longer than the cross-over point were significantly greater than the volumes of the controls. These tests were performed separately for each ROI with the cross-over points of each ROI identified from the linear regressions. All of these tests were significantly different (all p<0.05).

#### Independence between use and abstinence effects

We tested whether the areas shown to have altered volumes associated with years of use were also observed to change with abstinence. We performed correlations for abstinence effects in those areas that showed years of use effects (and *vice versa*). For all clusters, only two, the right precuneus and left cuneus clusters identified initially as showing positive correlations with abstinence (p<0.05) also showed significant negative correlations with years of use (p<0.05).

## Discussion

The present results are some of the first to examine grey matter volumes related to the length of cocaine use and abstinence in a population of former cocaine addicts. We observed several regions displaying decreased GM with increasing years of use. Although these results are necessarily correlational, they suggest a cumulative effect of cocaine use wherein the longer the period of substance use the lower the grey matter volume [22]. That these effects were observed in abstinent users is consistent with prior reports of GM deficits in alcoholism that last from 6–9 months to more than a year or, in some reports, up to at least 6 years following abstinence [42]–[44]. Similarly, decreased GM as a function of years of use of heroin [6], [45], [46] and cocaine [15] have previously been reported. Conversely, increased GM as a function of years of use was also observed in the cerebellum, bilateral precentral gyrus (both effects discussed below) and also in the perigenual region of the cingulate gyrus associated with affective processing [47]. This may be a consequence of repeated cocaine use blunting responses in regions important to emotional regulation [48]. Alternatively, given that emotional reactivity has been implicated as a factor modulating vulnerability to drug abuse [49], this may have been a preexisting factor that served to increase the likelihood of the development and prolongation of drug abuse.

If addiction can be characterized as a loss of self-directed volitional control [22], abstinence and its maintenance may be characterized by a reassertion of these aspects of executive function [24]. Current cocaine users demonstrate reduced GM in brain regions critical to executive function, such as the anterior cingulate, lateral prefrontal, orbitofrontal and insular cortices [6]–[11]. In contrast, the group of abstinent CD users reported here show elevations in GM as a function of abstinence duration that exceeds control levels after 36 weeks, on average, of abstinence. One possible explanation for this is that abstinence may require reassertion of cognitive control and behavior monitoring that is diminished during current cocaine dependence [11], [50], [51]. We, and others, have previously hypothesized that drug abusers may develop increased cerebellar activity to compensate for reduced prefrontal activity in tasks demanding elevated levels of cognitive control [52], [53] and that this may play a role in maintaining abstinence [24]. Reassertion of behavioral control may produce a practice-related expansion [54] in GM regions such as the anterior insula, anterior cingulate, cerebellum, and dorsolateral prefrontal cortex and is consistent with our previous reports of elevated activity levels, compared to controls, in long-term abstinent substance users [24], [55]. A viable alternative, given the cross-sectional nature of the data, is that the differences in GM volumes preceded abstinence and the relationship with abstinence duration indicates that those with greater volumes in these regions are more likely to maintain abstinence for longer. A small, but growing, body of literature has begun to examine this possibility in users of several substances as assessing baseline predictors, such as grey matter volume, may provide an indication of what might be different from the onset of abstinence in those who maintain abstinence. In the case of alcohol, gray matter volume in the parietal-occipital sulcus, medial and right lateral prefrontal cortex [56] and brain regions critical to behavioral control and reward processing [57], [58] have been shown to predict likelihood of relapse and successful abstinence. Similarly, grey matter volume in cortical and subcortical regions measured prior to cessation has been shown to be predictive of treatment outcome in smokers [59]. To our knowledge, no similar morphometric analyses of grey matter in users of stimulants, such as cocaine, have been performed. However, a variety of functional activation studies have shown that activation levels in brain regions associated with behavioral control, interoception and reward valuation show promise as predictors of treatment outcome in methamphetamine [60] and cocaine users [61]–[64]. We have previously investigated the integrity of white matter in the same cohort of CD users as reported here [25]. That study identified a dissociation of disease and abstinence effects that are consistent with the results reported herein. For example, the prefrontal changes reported here may complement white matter changes we previously observed in the longitudinal fasciculus [25]. It should be noted, however, that our previous DTI study did not include tractographic analyses so we cannot be certain that the grey matter changes reported here are linked to the white matter changes that we have previously reported. Future studies that investigate both grey matter and tractographic differences that may be related to duration of abstinence and length of use are required to resolve this ambiguity. Ultimately, adjudicating between these alternatives, namely that the volume differences reported herein arose as a consequence of abstinence or predated and facilitated abstinence, requires large-scale longitudinal studies. Nevertheless, both interpretations of the present data identify elevated levels of volume in regions that underlie cognitive control as characteristic of successful abstinence.

Impulsivity has been identified as a risk factor for the development of substance use disorders wherein individuals displaying higher levels of impulsivity are prone to both experimentation with and misuse of illicit drugs [65], [66]. Additionally, substance use may influence maladaptive behaviors through either acute effects (such as through action on the midbrain dopamine system [67], [68]), or as a consequence of prolonged drug use. For example, acutely, drugs may lead to impaired inhibition [50] and altered risky-choice behavior [51], [69]–[71]. Continued use may result in escalation of use and subsequent dependence, possibly by altering the neural substrate of performance monitoring [72] and stimulus-reward processing brain systems [73], amongst others. A common observation in trait impulsiveness is elevated motor activity [74]. The observation of elevated GM reported in bilateral precentral gyrus with years of use may be significant insofar as it may reflect elevated environmental exploration on the part of the addict to procure the abused substance [75]. Indeed, such an hypothesis is consistent with reports of increased GM in motor cortex with the acquisition of complex motor skills [76].

Left and right inferior frontal gyrus and right anterior cingulate have been identified as key loci underlying response inhibition [77]–[81] and are associated with impaired cognitive control in current addicts [82] and heavier, prolonged substance abuse [83]. As noted above, impaired behavioral inhibition is one of the defining characteristics of drug addiction. The observation of reduced GM with years of use in these regions may reflect the cumulative effect of damage caused by prolonged usage. Previous VBM studies of cocaine addicts have observed reduced GM in cerebellum [12] and have suggested that this may reflect the cumulative effect of cocaine-induced oxidative stress and vasoconstriction [12]. Furthermore, the region of reduced GM is located in a lobule of the cerebellum with many reciprocal connections to prefrontal cortex [84], [85]. This may contribute to an inability to moderate behavior notwithstanding any possible negative consequence it may have [22], [86], [87], and thus contributing to continued drug abuse. Alternatively, these effects may have been preexisting and constitute an endophenotype for impaired behavioral control that may have contributed to the development of drug abuse [11]. It should be noted that we also observed regions displaying increased GM with abstinence in bilateral cingulate gyri that did not overlap with those showing decreased GM with years of use. This suggests that the brain is capable of compensating in response to changes in demands, such as the maintenance of abstinence [54], [76].

The present results are tempered by some limitations. A fuller characterization of the subjects would be of value in order to assess the psychological consequences of the observed structural changes. In addition, the CD group reported here included individuals who were dependent on alcohol and heroin. While polydrug use of this sort is representative of the CD population, it raises the possibility that the effects reported here could be influenced by these other drug dependencies. Future studies might aim to resolve this ambiguity by recruiting a purely cocaine dependent cohort or a larger sample of polydrug abusers which would facilitate analyses to explore independent and interactive drug use effects. Additionally, future studies should aim to determine whether the number of attempts at abstinence has any bearing on GM change. Finally, consistent with most human clinical studies, it is not possible to address the etiology of the changes reported here. That is, we cannot say with certainty that they arose as a consequence of cocaine consumption or predated it. Notwithstanding this ambiguity, the present results demonstrate a dissociation between the effects of prolonged addiction and extended abstinence. The dissociation between regions showing alterations in grey matter with increased years of use and those altering with increased abstinence suggests that recovery is not simply a reversal of the process of disease. Rather it suggests an asymmetry between the two wherein cortical regions critical to behavioral control may serve as a biomarker of successful abstinence. Furthermore, these systems may be apt for targeting during treatment, such as with mindfulness-based approaches [88] that have been shown to modulate both function and structure of some of the regions reported here [89]–[91]. This may ultimately lead to decreased relapse and increase the likelihood of prolonged, successful abstinence.
